# Water-Holding Capacity, Ion Release, and Saturation Dynamics of Mosses as Micro-Scale Buffers Against Water Stress in Semi-Arid Ecosystems

**DOI:** 10.3390/plants14172728

**Published:** 2025-09-02

**Authors:** Serhat Ursavas, Semih Edis

**Affiliations:** Faculty of Forestry, Department of Forest Engineering, Cankiri Karatekin University, 18200 Cankiri, Turkey; semihedis@karatekin.edu.tr

**Keywords:** moss saturation dynamics, water holding capacity, moss hydration, ecohydrology

## Abstract

Mosses are key players in semi-arid ecosystems; however, the functional roles of mosses on hydrologic buffering and water quality have hardly been assessed. In the present study, the water storage, saturation dynamics, and ion release experiment of a set of four moss species (*Hypnum lacunosum*, *Homalothecium lutescens*, *Dicranum scoparium*, and *Tortella tortuosa*) was performed by a more simplified immersion and drainage procedure with water chemistry analyses. All species reached a sorption equilibrium between 10 and 20 min, with pleurocarpous taxa retaining 20–35% more water than acrocarpous species and possessing water-holding capacities (WHCs) between 300% and 700% of dry weight. Species-specific differences in water chemistry (pH, EC, and TDS) were observed: *Tortella tortuosa* presented the greatest ionic flux, and *Hypnum lacunosum* presented little variation in pH and electrical conductivity. These findings imply that the mosses operate as micro-scale buffers regulating both water quantity and water quality, and thereby the soil stability, infiltration, and drought resilience. The combined hydrological and biogeochemical view offers a novel understanding of bryophyte ecohydrology and highlights the significance of mosses in the practice of watershed management and climate-change mitigation.

## 1. Introduction

Mosses, liverworts, and hornworts (hereafter bryophytes) are a group of primitive non-vascular plants with crucial ecological roles despite their diminutive size and simple architecture. They control local micrometeorological conditions, for example, by reducing soil surface temperature through shading and evaporative cooling and by increasing near-surface humidity via water retention, protecting against soil erosion, promoting water infiltration, affecting nutrient cycling, and creating microhabitats that increase biodiversity [1,2,3,4]. Their roles in ecostability and resilience are especially important in semi-arid ecosystems with high variability in water availability and increasing drought intensity under global climate change conditions [5,6]. When mosses are the dominant ground covers, precipitation can be intercepted by mosses, surface runoff can be reduced by as much as 91%, and infiltration can be increased by about 85%, and soil erosion can be nearly eliminated when they cover bare soil, which is conducive to the retention of water in ecosystems and the protection of soil resources [7,8]. These hydrological and ecological functions are underpinned by distinctive morphological and physiological characteristics. Structural features such as marginal capillary leaves, papillae, and hyaline cells promote rapid external water absorption and water distribution, while physiological strategies such as desiccation tolerance and rapid rehydration ensure persistence during extended dry periods [9,10,11]. Consequently, moss mats may buffer short-term shifts in soil moisture dynamics, offer stable microsites for soil invertebrates and soil microorganisms, and could facilitate seedling establishment and vegetation regeneration in water-limited systems [12,13,14]. These traits make mosses key sensors of soil–plant–atmosphere interactions in ecosystems prone to climatic extremes.

In addition to providing a physical structure for water capture and storage, mosses also have effects on water chemistry via ion release. During hydration events, bryophytes can release or take up ions that may influence the pH, electrical conductivity (EC), and total dissolved solids (TDS) in surface and infiltrating waters [15,16]. Some species of peat moss (e.g., Sphagnum) are acidophytic since they acidify their habitat through hydrogen ion release (via the exchange of protons with cations such as Ca^2+^ and Mg^2+^), while others increase the concentration of divalent cations such as calcium and magnesium, thus changing the ionic composition and nutrient availability [17,18]. These chemical associations have implications for monitoring nutrient cycling and atmospheric deposition on a wider scale; however, mosses will effectively take up and release trace elements, heavy metals included, depending on environmental exposure [19,20]. Yet, although the role of these processes is crucial, a limited number of studies have quantified ion release under laboratory-controlled conditions or investigated its association with the species-specific structural traits and hydration dynamics [21].

The ecological importance of mosses is also emphasized in the face of climate change, leading to the increased frequency and intensity of droughts in many semi-arid systems. Mosses, as poikilohydric life forms, must keep in balance with moisture oscillations in their habitat [5,11]. Although many species of living organisms are capable of desiccation tolerance and can quickly recover from desiccation upon re-wetting, severe or repeated drought stress can lead to declines in their water-holding capacity (WHC) and influence local water balances, thus preventing them from performing roles in carbon sequestration and nutrient retention [22,23,24]. In ecosystems, such as peatlands and forests, where moss-rich layers are key components of carbon stocks, a drought-induced response in mosses may exert significant controls over the greenhouse gas balance and soil vitality [25,26]. Similarly, the WHC of mosses to temporarily store and slowly release water serves to make them micro-scale buffers of climate-driven water stress, which could possibly act to dampen the effects of water stress and enhance ecosystem resilience [6,14]. However, despite this recognition, there continue to be methodological impediments to quantifying moss WHC, rates of saturation, and concomitant ion release. Existing methods vary from rainfall simulation to long-term immersion and are frequently conducted with non-standardized immersion times and analytical procedures, which makes it difficult to compare different studies and to combine hydrological and chemical research findings [11,27]. Additionally, the relationship between water absorption dynamics, ion release patterns, and their interaction has not been extensively studied, pointing to a knowledge gap between moss’s functional traits controlling water quality regulation and moss’s functional traits determining micro-scale hydrology.

This research gap is explored in the present study with two goals: (i) to design a simple and fast laboratory method that can be replicated for quantifying moss WHC and its saturation dynamics in vitro, and (ii) to assess the effect of the corresponding ion release (pH, EC, and TDS). Specifically, we examine four ecologically different moss species (*Dicranum scoparium* Hedw., *Hypnum lacunosum* (Brid.) Hoffm. ex Brid., *Homalothecium lutescens* (Hedw.) H.Rob., and *Tortella tortuosa* (Schrad. ex Hedw.) Limpr.), combining morphological, hydrological, and chemical approaches to provide insight into their function as micro-scale buffers of water stress in semi-arid systems. The species demonstrate a range of ecological strategies, including shade-tolerant, forest-floor mosses such as *D. scoparium* and *H. lacunosum*, as well as xerophytic taxa found in open calcareous environments like *T. tortuosa* and *H. lutescens*. This diversity establishes an ecological gradient useful for assessing water-holding and ion release characteristics [3,11,28,29]. These findings advance the science of bryophyte ecohydrology and have implications for the control of water quality, ecosystem stability, and adaptation to climate change.

## 2. Results

### 2.1. Descriptive Statistics

Upon assessment following weight measurements and laboratory water quality analyses, it was determined that the pH level was 4.65, resulting in an acidified environment for the saturated species. An examination of the standard deviation of water-saturated weights indicated discrepancies among the measurements. Furthermore, the coefficient of variation (CV) analysis revealed significant variability in the saturated weight, electrical conductivity (EC), and total dissolved solids (TDS) values (Table 1).

### 2.2. Saturation Kinetics

**Figure 1:**
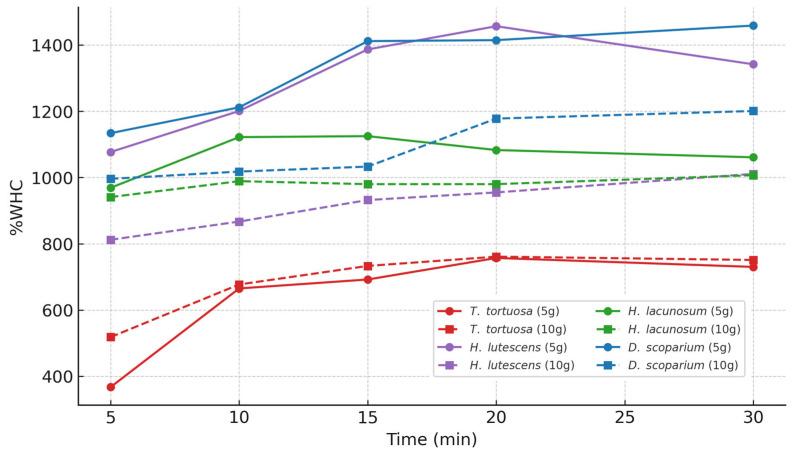
WHC curves and minimal duration required to reach peak saturation for each moss species at 5 g and 10 g sample masses. Different lowercase letters indicate significant differences among species and masses (Tukey HSD, *p* < 0.05).

As can be seen in Figure 1, the time profile of the water-holding capacity of all species showed a rapid water uptake in the first 10–30 min. The highest maximum WHC value was found for *H. lutescens* (≈1464%), followed by *D. scoparium* (≈1461%), *H. lacunosum* (≈1127%), and *T. tortuosa* (≈758%). *T. tortuosa* always had a lower WHC as compared to the other species under saturated conditions. Comparisons between 10 g and 5 g (Figure 1) showed that smaller samples equilibrated faster, whereas ultimate WHC values were similar.

Temporary decreases in the water-holding capacity were recorded in *H. lacunosum* after 15–20 min, and in 5 g samples, these were most evident. The effects of sample mass on saturation kinetics were, in general, small. The mass-independent, lowest WHC time was observed in *D. scoparium*, *H. lacunosum*, and *T. tortuosa*. Conversely, *H. lutescens* took more time to reach saturation levels at a sample mass of 10 g compared to 5 g. *D. scoparium* required approximately 30 min to reach the corresponding saturation (Table 2).

**Figure 2:**
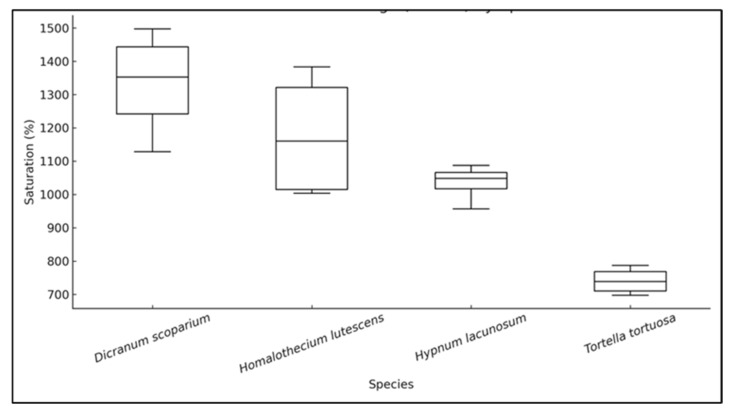
Maximum WHC percentage at 30 min for different moss species (*H. lacunosum*, *H. lutescens*, *D. scoparium*, and *T. tortuosa*).

The sample weight did not affect the minimum time to WHC in *D. scoparium*, *H. lacunosum*, and *T. tortuosa*. In *H. lutescens*, it increased in proportion with the sample weight. The species curves illustrating changes in the dynamics of saturation (water-holding capacity) over time indicate the effects of the sample weights (5 g and 10 g) and interspecific differences. Means and standard deviations were also calculated, where data points representing a single observation (n = 1) are depicted as open circles. From these measurements, the results indicate that some species exhibit a faster WHC, and weight variation may produce different effects across species (Figure 2).

The analysis of 0.5 g and 1 g samples revealed proportional increases that were comparable to those of 5 g and 10 g samples. The peaks of the smaller sample weights were reached more rapidly than those of the larger sample weights. Nevertheless, the magnitude and direction (positive or negative) of mass effects varied among species. Except for *D. scoparium*, 5 g samples absorbed water at a significantly faster rate than 10 g samples. Uptake rates were lower in larger (10 g) samples, consistent with the reduced capillary continuity. In *H. lutescens*, water retention was greater in 10 g than in 5 g samples. The responses of these species were not particularly sensitive to sample size, as the results of the study in *H. lacunosum* and *T. tortuosa* indicate no significant differences between treatments of 5 g and 10 g.

### 2.3. Species Differences in Water-Holding Capacity

Moss species show a significant variation in WHC. Species exhibited considerable differences in WHC (ANOVA: F(3,12) = 14.63, *p* < 0.001). The species effect size was determined to be η^2^ = 0.79. Pairwise comparisons indicated that *T. tortuosa* exhibited a markedly lower WHC relative to all other species (Cohen’s d: *T. tortuosa* vs. *H*. *lutescens* = −3.11; *T. tortuosa* vs. *H. lacunosum* = −5.97; and *T. tortuosa* vs. *D*. *scoparium* = −4.96) (Table 3).

Furthermore, the sample mass influenced WHC in certain species. Five-gram samples of *D. scoparium* exhibited a greater WHC at 5 g, but its water-holding capacity diminished as the sample weight increased. Conversely, *H. lutescens* retained more water at larger sample masses. The water-holding capacity in *H. lacunosum* and *T. tortuosa* was largely sample-weight independent (Figure 2; Table 3).

Analysis of the various sample weights of different moss species revealed that *D. scoparium* exhibited the highest water-holding capacity (WHC) value of 5 g. The water-holding capacity diminished as the sample weight increased. The water-holding content in *H. lacunosum* and *T. tortuosa* was sample-weight independent. The sample biomass for *H. lutescens* retained more water.

### 2.4. Physicochemical Properties (EC, TDS, and pH) of Retained Water

Consequently, parameters such as electrical conductivity, total dissolved solids (TDS), and pH were assessed in water samples where the mosses retained their saturation for 24 h. In this context, the results of the Tukey HSD analysis, conducted to evaluate and compare the disparities in water properties such as pH, EC, and TDS among different moss species, revealed significant variations between the species. Based on EC values, *T. tortuosa* exhibited significant differences relative to the other three species (*p* < 0.001). No variation was observed among the other species regarding EC levels. A comparable outcome was noted in TDS values, revealing that *T. tortuosa* exhibited significantly lower TDS levels relative to the other species. Significant differences in pH values were observed among all moss species (*p* < 0.05). The significant disparity between *T. tortuosa* and *D. scoparium* indicated that both species experienced distinct environmental conditions (Table 4).

After 24 h of incubation, the pH decreases, and the electrical conductivity increases. These results validate the spatial clustering of salt stress in the dendrogram, with *T. tortuosa* placed at a separate node with a relatively low ion efflux and *H. lutescens* consistently showing the highest pH and ion concentrations.

### 2.5. Multivariate Analyses

**Figure 3:**
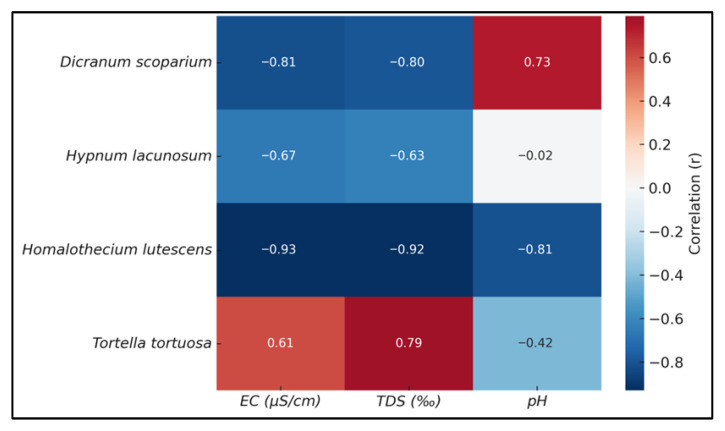
Correlation coefficients (Pearson r) between WHC by species and EC, TDS, and pH.

The variations among species regarding the relationships between WHC, ion release (EC, TDS), and pH are substantial. In *H. lutescens* and *D. scoparium*, a strong negative correlation was found between WHC and both EC (r = −0.93 to −0.81) and TDS (r = −0.92 to −0.80). In *H. lacunosum*, a negative correlation was observed with the electrical conductivity (EC) and total dissolved solids (TDS) (WHC % between EC: −0.67; WHC % between TDS: −0.63), while no significant relationship was identified with pH. *T. tortuosa* exhibited a distinct pattern in which ion release was positively correlated with the WHC (r = 0.61 for EC; r = 0.79 for TDS). A moderate negative correlation was observed with pH, quantified at −0.42 (Figure 3).

**Figure 4:**
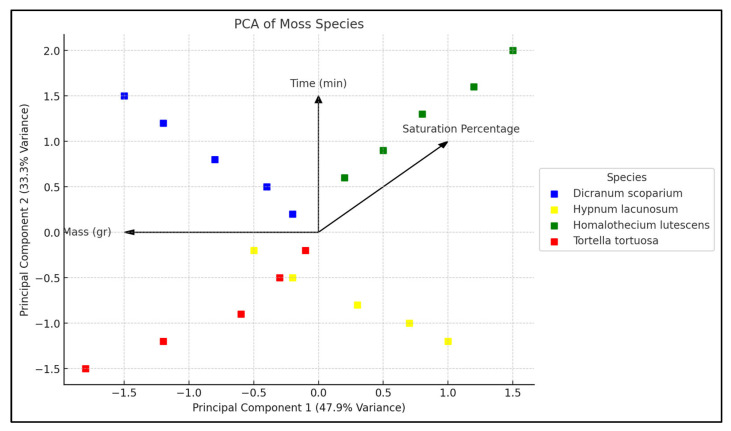
Scatter plot of Principal Component Analysis (PCA), illustrating the distribution of moss species.

The PCA results indicate that PC1 and PC2 account for the majority of the variation, with PC1 at 47.9% and PC2 at 33.3%. The total variance is 81.2%. The loadings were greatest for WHC on PC1 (70.7%), which was the axis where the explanation of variation was most significant for that metric. The focus on weight (64.1%) and duration (76.7%) was significant on PC2. The *T. tortuosa* species exhibits a cumulative signal in both components, suggesting a significant divergence in water retention. This study establishes a foundation for understanding the key variation points of the species-level water-holding capacity, along with the parameters that define those variations (Figure 4).

## 3. Discussion

### 3.1. Water-Holding Dynamics and Species Differences

Our data indicated that the four mosses analyzed became fully saturated after 10–20 min of exposure, with some differences in light WHC between the species. *H. lacunosum* and *D. scoparium* reached the maximal WHC at the earliest time and *T. tortuosa* at the latest. In addition, the relative WHC of the pleurocarpous species (particularly *H. lutescens and H. lacunosum*) was generally greater than that of *T. tortuosa*, while *D. scoparium* exhibited higher WHC values compared to *H. lacunosum*. These results agree with other studies that reported that pleurocarpous mosses can absorb water more rapidly and achieve a higher WHC than acrocarpous mosses [30,31]. This difference is attributed to their spreading shape in the horizontal direction and a larger contact surface area. However, in our dataset, *D. scoparium* exhibited a higher WHC than *H. lacunosum*, which may be attributed to its morphological traits, such as its dense mat formation and recurved leaves, enhancing its water storage capacity.

These differences can mostly be explained by morphological traits. Pleurocarpous species, characterized by creeping shoots and overlapping leaves, enhance outward capillary conduction [3,11]. In contrast, acrocarpous mosses are denser and rely mainly on internal water storage. *T. tortuosa*, with its upright growth form typical of open habitats, showed slower saturation and a more gradual increase in WHC in our dataset. This pattern shows how plants have adapted to exposed microsites, where tightly packed leaves and thicker cuticles help them survive quick wet–dry cycles but make it harder for them to take in water right away [32].

The presence of hyaline cells in pleurocarp mosses, along with the concave structure and branching pattern observed in their cross-sections, enhances the absorption and conduction of water [33]. These structures enable *H. lacunosum* and *H. lutescens* to achieve water-holding capacity more rapidly. The non-layered leaves of *D. scoparium* restrict the swift attainment of this capacity. Species-specific morphological traits are believed to primarily account for the observed variations in water-holding capacity (WHC) [34,35].

The WHC duration recorded in this study (10–20 min, 300–700% of dry weight) was similar to previous studies on mosses distributed in semi-arid regions [36,37]. Typical values of moss’s WHC in semi-arid ecosystems range between ~108% and 5000%, expressed as a percentage of dry matter [8,38]. This range aligns with our measured values (300–700% DW). *T. tortuosa* showed the lowest values, while *H. lutescens* had the highest within our dataset, in accordance with their structural and physiological features. Our findings on these four species represent, to the best of our knowledge, the first report of the WHC measured directly [39]. Direct WHC measurements are widely lacking for many moss species, such as *T. tortuosa* and *H. lutescens*, a trend that reflects trait voids observed at larger scale moss ecologies [40]. Thus, open habitat species generally hydrate longer than bryophyte mat species, confirming the longer WHC time and slower drying of *T. tortuosa* [3]. These interspecific differences demonstrate the importance of moss diversity in buffering moisture and water availability at the micro-scale in semi-arid environments [5,41].

### 3.2. Ion Release and Ecohydrological Implications

Our results show that varying amounts of species released different amounts of dissolved water, EC, and total dissolved solids (TDS). For example, *H. lacunosum* had only a minor effect on water chemistry and maintained an almost neutral pH value. This can be explained by the hydration and desiccation cycles of mosses. Features such as increased membrane porosity and adsorption–desorption dynamics significantly affect the water chemistry, especially pH, EC, and TDS, as previously documented in the literature [9,42,43].

The specific characteristics of ion release for each species are thoroughly documented. For instance, Sphagnum mosses lower the pH of their surroundings by exchanging calcium and magnesium ions for protons. Conversely, species such as *Thuidium delicatulum* (Hedw.) Schimp. and *Brachythecium rivulare* Schimp. have the ability to increase the concentration of divalent cations in the surrounding water [44,45,46]. Although Sphagnum and aquatic mosses differ ecologically from semi-arid taxa, they provide established examples of ion release processes that contextualize our findings. The elevated ion release noted in *T. tortuosa* indicates greater water content or accelerated desorption during hydration events [42]. The relationship between water retention and ion release also provides context for our findings. Bryophytes with a higher water capacity have a tendency to maintain the integrity of their ions and reduce the loss of solutes during rapid wet–dry cycles [47,48]. Our observation that *H. lacunosum* possessed both a faster WHC and a smaller release of ions suggests that the efficient retention of ions is associated with its pleurocarpous growth form. In contrast, the slower WHC *T. tortuosa* released a larger amount of ions, which supports the general association between the WHC and stability of ions [49].

Beyond pH control, mosses have a significant effect on the surrounding environment in terms of calcium and magnesium. Studies have demonstrated that taxa like *T. delicatulum* and *B. rivulare* can increase the concentration of divalent cations in water by as much as 36%. This effect was reported in Canadian lakes and other habitats that are calcicolous [45,46,50]. These results show how important moss-covered areas are as small-scale buffers that let more water soak in, reduce surface runoff, and keep soil stable in dry ecosystems [51,52,53]. Their ability to tolerate drying out and quickly rehydrating also helps nutrients move around and keeps the ecosystem strong during dry spells [54].

### 3.3. Methodological Considerations and Future Research

In this study, the water-holding capacities (WHCs), saturation kinetics, and ion leakages of mosses were investigated by a simplified immersion-filtration method. Although the method is practical and reproducible, it is not fully compatible with classical techniques used in eco-hydrology. Hydraulic conductivity, water release, pore size, and evaporation experiments are more detailed but limited for rapid species comparisons [55,56,57]. The simultaneous assessment of water and ion release is increasingly important in understanding bryophyte ecophysiology. Findings on drying–wetting cycles and cation exchange capacity are directly related to nutrient cycling and physiological responses [47,58,59]. In this study, the WHC was correlated with pH, EC, and TDS. Monitoring the temporal variations in ion fluxes with continuous measurement techniques and correlating them with microclimatic conditions is important for future research. Furthermore, WHC estimates are affected by sample mass and size, making sampling design a critical element [27,60]. Larger samples can better reflect structural diversity; they may increase variability in results. In our research, there was no significant difference between the 5 g and 10 g samples; this suggests a practical and significant composition. Adaptive protocols for sampling that take into account the habitat’s morphologies may contribute to future studies.

## 4. Materials and Methods

### 4.1. Materials

This study utilized four bryophyte species exhibiting distinct morphological growth forms. Two acrocarpous mosses (*Dicranum scoparium* and *Tortella tortuosa*) and two pleurocarpous mosses (*Homalothecium lutescens* and *Hypnum lacunosum*) were selected for analysis (Figure 1). These species are common in semi-arid forest ecosystems and vary in their structure, which reflects different possible effects on their WHC, saturation dynamics, and ion release characteristics.

**Figure 5:**
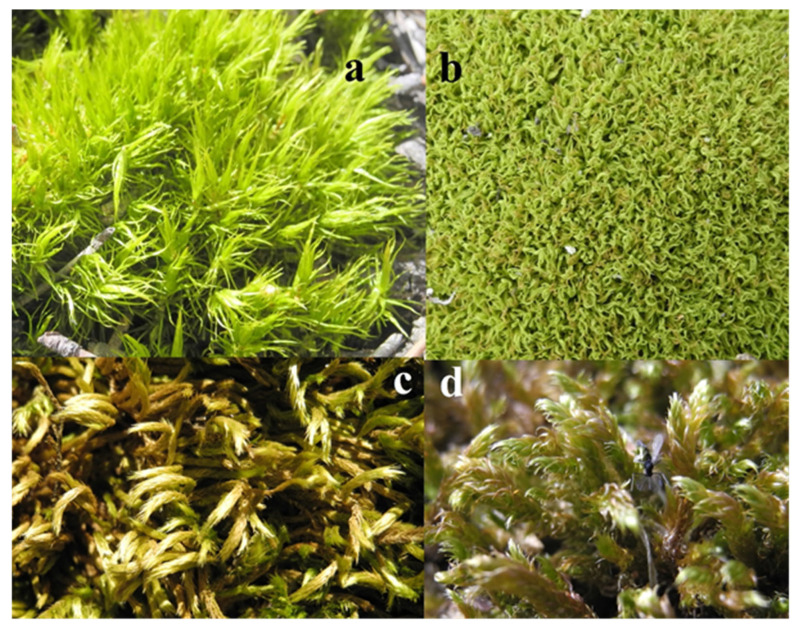
Selected moss species categorized as acrocarpous mosses ((**a**) *D. scoparium*, (**b**) *T. tortuosa*) and pleurocarpous mosses ((**c**) *H. lutescens*, (**d**) *H. lacunosum*).

Specimens (Figure 5) were taken from the Eldivan Mountain, Çankırı Province, Central Anatolia, Türkiye, which has a continental semi-arid climate. The monthly average temperature varies from −0.4 °C in January to 23.1 °C in July (long-term climate data 1929–2024); the corresponding annual mean temperature is 11.4 °C. Annual average rainfall is 415 mm, with the maximum in May (57.1 mm) and minimum in August (18.4 mm). The average annual sunshine duration is 6.4 h/day, and the number of rainy days is about 106 days per year, with a peak during spring and late autumn. Extreme temperature variations were observed, with absolute minimum and maximum values of −25.0 °C and 42.4 °C, respectively, during the observation period [61].

### 4.2. Methods

**Figure 6:**
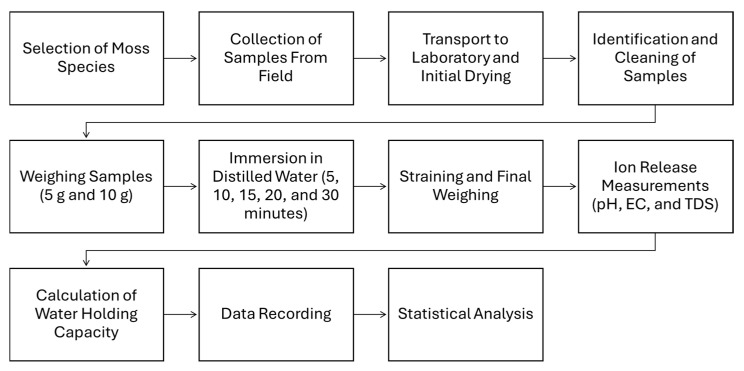
Flowchart outlining the study design and methodology.

In all trials, the WHC was assessed by determining the highest volume of water retained per gram of dry bryophyte mass. There is currently no universally accepted defined approach for evaluating WHC, resulting in variations in methodology across different research [62]. Subsequent to the taxonomic identification conducted in the laboratory for selection, field samples were collected, followed by laboratory procedures including sample preparation, identification, weighing, *WHC*, and concluding with a statistical analysis conducted in the office (Figure 6). Consequently, each phase of the experimental process has been executed meticulously to ensure systematic and stepwise progression.

The approach used in this study builds upon established WHC measurement techniques employed in bryophyte research (e.g., [15,48]). While components such as immersion timing and weight tracking are not individually novel, their combination in a time-structured protocol linked to electrical and chemical water quality parameters offers an operationally simple and repeatable method suited for comparative ecological analysis in semi-arid forest conditions. The focus here lies in practical adaptation rather than methodological invention.

#### 4.2.1. Field Sampling

The habitat is mainly characterized by stands of *Pinus nigra* with open patches. Samples of *D. scoparium*, *H. lutescens*, and *H. lacunosum* were obtained from under *P. nigra* canopy layers and *T. tortuosa* from relatively open habitats and exposed soils, reflecting its xerophytic ecological preference. The four species of moss were chosen to represent different ecological strategies that are typical of dry regions with precipitation deficits. *D. scoparium* is a moss that forms tufts in order to absorb moisture from the air and the soil; this species is common in forest soil that is characterized by acidity and supports a high moisture tolerance [11,28]. *T. tortuosa* typically inhabits limestone rocks and open, exposed areas, forming compact cushions that minimize the loss of water during dry periods [29]. *H. lacunosum* is associated with grasslands that have a high degree of calcification; it forms floating mats that consume dew and provide moderate drought resistance [3,63]. *H. lutescens* thrives in dry, bright grasslands that are covered with calcareous soil; there, it forms dense sheets and exhibits high physiological resilience, including the stability of the photosystem II in heat and drought conditions [64]. These ecological differences justified their selection as the model species to contrast the capacity for water storage and the release of ions. Additionally, these taxa are common in the regionally diverse forests and pastures of Anatolia, which makes them both environmentally significant and functionally distinct, despite not being of immediate concern.

#### 4.2.2. Sample Preparation

After collection, the specimens collected were transported to the laboratory in perforated paper bags to minimize condensation and microbial degradation. After air-drying, the samples were weighed using a precision analytical balance. Some foreign particles (dirt, wood, and litter) were picked by hand during the collection. The species were identified by stereomicroscope and regional floras [65,66].

#### 4.2.3. WHC Measurement

Prior to experimentation, moss samples were thoroughly cleaned of extraneous materials such as sticks, stones, soil particles, and other plant fragments. Each moss species was air-dried for 48 h in a shaded, well-ventilated environment to allow for moisture stabilization without compromising the integrity of cellular structures. Although oven-drying at 60–70 °C is standard in absolute dry weight protocols, it was not employed in this study to prevent cellular degradation and preserve natural morphology, which is critical for simulating realistic hydration dynamics.

Subsequently, air-dried samples were weighed using a precision analytical balance (±0.001 g). For each species, ten biological replicates were prepared for each of two dry-weight classes (5 g and 10 g), resulting in a total of 80 experimental units (4 species × 2 weight classes × 10 replicates). Each sample was immersed in 0.5 L of distilled water at room temperature using a stainless-steel strainer to ensure uniform submersion without mechanical disturbance. Immersion durations were set at 5, 10, 15, 20, and 30 min to assess WHC over time.

At the end of each interval, samples were allowed to drain for five minutes on a mesh platform to remove superficial water, after which the final weights were recorded. The water retained by the moss was determined by calculating the difference between the saturated weight and air-dry weight.

It is important to note that relative water content (RWC), a commonly used physiological parameter, was not explicitly calculated in this study. Instead, the WHC was used as a proxy for evaluating the relative water content (RWC) of mosses. This approach is consistent with previous bryological studies where RWC estimation was not feasible under rapid immersion protocols [15,48]. Nevertheless, future studies are encouraged to incorporate RWC under controlled environmental conditions to enhance comparability.

To determine the minimum duration required for each moss species to achieve WHC, we calculated the time needed to reach 98% of its maximum WHC (Equation (1)). This approach is consistent with methodologies commonly applied in studies of bryophyte hydration dynamics [67].

%WHC = (Ws − Wd)/Wd × 100(1)

where

%WHC: Water-holding capacity (saturation percent);

Ws: Water saturated weight;

Wd: Air-dry weight.

Then, the minimum WHC time for each moss species was identified as the duration nearest to the point of maximum saturation attainment.

#### 4.2.4. Ion Release Assay

Following the water retention experiment, separate sets of samples (also air-dried and rehydrated) were submerged in distilled water and maintained for 24 h to assess changes in water chemistry. Electrical conductivity (EC), total dissolved solids (TDS), and pH were measured using portable probes (Hanna Instruments, HI98129) following standard water quality procedures [68].

#### 4.2.5. Statistical Analysis

Effect size metrics (η^2^ and Cohen’s d) were used to supplement *p*-values by providing an estimation of the magnitude of treatment effects. ANOVA was followed by a post hoc Tukey HSD test to assess pairwise differences among species. Time-dependent WHC curves were evaluated using mean ± standard deviation, with points featuring single observations (n = 1) distinctly identified in the figures. Pearson correlation coefficients between WHC and pH, EC, and TDS were computed to produce a correlation heat map. In addition to the Pearson correlations, a partial correlation analysis was performed to control the effect of the species identity as a categorical factor. These analyses were performed to reveal distinctive patterns in the relationships between WHC and pH, EC, and TDS to account for the species’ differences. The species-induced variance was statistically removed in the generation of the correlation heat map. This led to a more accurate depiction of the associations among variables that were not taxon-dependent. Principal Component Analysis (PCA) was utilized to investigate multivariate relationships among species, sample mass, and hydration variables, resulting in PCA biplots that illustrate variable loadings. All analyses were conducted using R (Version 4.3.2; R Core Team) and Python (Version 3.11; pandas, seaborn, matplotlib, statsmodels, and scipy). Statistical significance was established at *p* < 0.05.

## 5. Conclusions

This research shows species-specific ion release, rapid water imbibition, and a pronounced WHC effect, exploring the very effective micro-scale water stress buffering capacity of mosses in semi-arid systems. All four species examined reached their WHC within 10–20 min, but their growth form had a significant impact on the WHC, with the pleurocarpous species (*H. lacunosum* and *H. lutescens*) storing 20–35% more water than the acrocarpous species (*D. scoparium* and *T. tortuosa*), with the WHC ranging from 300% to 700% of dry mass. Ion release data indicated marked functional differences, with *T. tortuosa* exhibiting the highest ionic release, which elevated leachate EC and TDS levels up to 25% higher than in the other species, whereas *H. lacunosum* maintained a near-neutral pH and low solute concentrations.

Collectively, these results demonstrate species-specific hydration and ion release patterns and a broader ecological relevance for semi-arid ecosystem functioning and resilience. Mosses moderate soil moisture, soil runoff, pH, and solute concentrations, and also aid in infiltration, erosion control, and the buffering of drought and water quality. The twofold hydrological and biogeochemical roles of the system underline the central contributions to soil stability, nutrient turnover, and regional drought resistance, particularly in a changing climate with more variable precipitation regimes and frequent drought periods. These findings show that mosses represent a nature-based solution that could be manipulated to manage watersheds and to restore and plan for climate adaptation.

Our results also demonstrate that ion release is species-selective and closely related to moss hydration traits, with follow-on effects for water quality management in semi-arid ecosystems. We suggest three methodological advances accordingly: (i) combining simplified WHC protocols with continuous, non-invasive moisture sensors, (ii) scaling ion flux measurements of WHC manipulations in mesocosms to field conditions, and (iii) using adaptive sampling designs that allow for morphologically different habitats. These methods will contribute to a better understanding of the eco-hydrological services of mosses and their adaptive capacity to regulate water quality and climate.

## Figures and Tables

**Table 1:** Descriptive statistics of variables.

Variables	Count	Mean	Std	Min	Max	Skewness	Kurtosis	CV (%)
Air dry weight (gr)	80.00	7.43	2.47	4.52	10.78	0.03	−1.97	33.32
Water saturated weight (gr)	80.00	79.67	25.81	22.70	126.65	−0.07	−0.88	32.40
Electrical Conductivity (µs/cm)	80.00	109.70	57.45	26.10	216.90	0.37	−1.21	52.37
Total dissolved solids (‰)	80.00	0.06	0.03	0.01	0.11	0.22	−1.29	54.83
pH	80.00	4.65	0.65	3.53	5.68	−0.24	−0.95	13.95

**Table 2:** Minimum duration (mins) required to reach ≥98% of maximum WHC for each moss species at two sample masses.

Species	Mass (gr)	Min Saturation Time (min)
*Dicranum scoparium*	5	30
*Dicranum scoparium*	10	30
*Hypnum lacunosum*	5	10
*Hypnum lacunosum*	10	10
*Homalothecium lutescens*	5	20
*Homalothecium lutescens*	10	30
*Tortella tortuosa*	5	20
*Tortella tortuosa*	10	20

**Table 3:** Mean water-holding capacity (%WHC) of moss species at different sample masses (5 g and 10 g). Different lowercase letters within a column indicate significant differences among species according to Tukey’s HSD test (*p* < 0.05).

Species	%WHC 5 g (±SE)	%WHC 5 g (±SE)
*Tortella tortuosa*	642 c	688 c
*Homalothecium lutescens*	1292 a	915 b
*Hypnum lacunosum*	1072 b	979 b
*Dicranum scoparium*	1326 a	1085 a

**Table 4:** Mean values (±SE) of pH, electrical conductivity (EC), and total dissolved solids (TDS) in water samples of moss species. Different lowercase letters within each column indicate significant differences among species according to Tukey’s HSD test (*p* < 0.05).

Species	pH (±SE)	EC (µS/cm) (±SE)	TDS (‰) (±SE)
*Tortella tortuosa*	4.9 b	40.0 a	0.020 a
*Homalothecium lutescens*	5.4 c	130.0 b	0.070 b
*Hypnum lacunosum*	5.2 b	120.0 b	0.065 b
*Dicranum scoparium*	3.9 a	110.0 b	0.060 b

## Data Availability

The data generated from field sampling and laboratory measurements in this study are not publicly available because they are part of an ongoing research project but are available from the corresponding author upon reasonable request.
